# Assessment of Thoracic Radiographic Alterations in Dogs with Heartworm and Their Correlation with Pulmonary Hypertension, Pre- and Post-Adulticide Treatment

**DOI:** 10.3390/ani14172551

**Published:** 2024-09-02

**Authors:** Soraya Falcón-Cordón, Yaiza Falcón-Cordón, Noelia Costa-Rodríguez, Jorge Isidoro Matos, José Alberto Montoya-Alonso, Elena Carretón

**Affiliations:** Internal Medicine, Veterinary Medicine and Therapeutic Research Group, Faculty of Veterinary Medicine, Research Institute of Biomedical and Health Sciences (IUIBS), Universidad de Las Palmas de Gran Canaria (ULPGC), 35016 Las Palmas de Gran Canaria, Spain; soraya.falcon@ulpgc.es (S.F.-C.); yaiza.falcon@ulpgc.es (Y.F.-C.); noelia.costa@ulpgc.es (N.C.-R.); jorge.matos@ulpgc.es (J.I.M.); elena.carreton@ulpgc.es (E.C.)

**Keywords:** vector-borne disease, *Dirofilaria immitis*, pulmonary hypertension, radiographic indexes, image diagnosis, echocardiography, veterinary diagnosis, dogs

## Abstract

**Simple Summary:**

Pulmonary hypertension (PH) is a common and severe complication in dogs infected with *Dirofilaria immitis*, often persisting even after treatment. This study aimed to evaluate the progression of PH in dogs by assessing changes in radiographic parameters and the Right Pulmonary Artery Distensibility (RPAD) Index before and after treatment. Parameters were measured on the day of diagnosis (Day 0), at discharge (Day 90), and six months post-discharge (Day 270). The results indicated that in non-hypertensive dogs, the RPAD Index significantly improved following treatment. In contrast, hypertensive dogs exhibited a persistently low RPAD Index throughout the study, indicating ongoing PH. Additionally, hypertensive dogs showed consistently elevated VHS, CrPA/R4, and CdPA/R9 ratios compared to non-hypertensive dogs. These findings underscore the persistence of PH despite treatment, suggesting that regular radiographic monitoring of VHS, CrPA/R4, and CdPA/R9 ratios is crucial for assessing and managing long-term outcomes in dogs with heartworm disease.

**Abstract:**

Pulmonary hypertension (PH) is a prevalent and severe complication in dogs infected with *Dirofilaria immitis*. This study aimed to elucidate the progression of PH by analyzing radiographic parameters and the Right Pulmonary Artery Distensibility (RPAD) Index at three key time points: diagnosis (day 0), discharge (day 90), and six months post-discharge (day 270). Fifty-two heartworm-infected dogs were divided into two groups: non-hypertensive and hypertensive. Radiographic measurements, including Vertebral Heart Size (VHS), CrPA/R4 ratio, and CdPA/R9 ratio, along with the RPAD Index, were assessed on Days 0, 90, and 270. Results indicated that, in Group A, the RPAD Index improved significantly from 42% on Day 0 to 43.16% on Day 90, with no significant change by Day 270 (42%). In contrast, hypertensive dogs exhibited a persistently low RPAD Index, averaging 17% throughout this study (*p* < 0.001). Radiographic parameters in hypertensive dogs showed continuous elevation compared to non-hypertensive dogs, with significant increases in VHS, CrPA/R4, and CdPA/R9 ratios on day 270 compared to day 0 (*p* < 0.05). The results confirmed that PH persisted in dogs with *D. immitis* after adulticide treatment, highlighting the importance of regular radiographic monitoring for assessing and managing long-term outcomes in dogs with PH during and after adulticide treatment. Continuous surveillance is thus essential for the effective post-treatment management of PH in dogs.

## 1. Introduction

Heartworm disease is caused by the nematode *Dirofilaria immitis*, with adult worms lodging in the pulmonary arteries and right ventricle and mainly affecting domestic and wild carnivores [1]. Pulmonary arterial hypertension (PH) is a frequent and generally severe condition in infected dogs; it is caused primarily by proliferative intimal changes driving irreversible structural damage to the vasculature, inflammation, loss of elasticity, and occlusion of the vascular lumen, leading to persistent PH [2,3]. In addition, embolization of dead heartworms contributes to the development and perpetuation of PH [1,4].

Previous authors agreed that severity and chronicity of pulmonary endarteritis can be assessed through the determination of PH [5,6,7,8]. To this aim, the use of echocardiography can provide valuable information for diagnosing PH based on indirect measurements, since right heart catheterization, which is the gold standard for diagnosing PH, is unacceptably invasive in routine procedures or in compromised patients [9,10].

Thoracic radiography is a valuable tool for identifying concurrent or underlying diseases in an individual patient and can also provide evidence supporting the presence of PH [9,11]. This imaging modality has the advantage that it is widely accessible for clinicians on a daily basis. Indeed, there are studies that have characterized the association of radiological and echocardiographic findings in dogs with PH [12,13]. In dogs with heartworm, thoracic radiographs may reveal right ventricular enlargement, main pulmonary artery dilation, and pulmonary artery tortuosity, although these findings are not specific [3,14]. Moreover, chest radiographs are useful in helping to estimate the severity and chronicity of heartworm disease [15]. More recently, a study in dogs with heartworm reported that evaluating certain thoracic radiographic measurements can serve as an objective preliminary screening when assessing whether complementary tests should be performed to evaluate the presence of PH [16].

Previous studies have shown that PH persisted in dogs after heartworm adulticide treatment for a minimum of 10 months following the completion of heartworm removal. The authors suggested that endarteritis may not be reversible after parasite elimination, highlighting the need for continued monitoring for PH after completion of adulticide treatment [8,17,18]. However, there is currently no research associating specific thoracic radiographic findings with the severity of PH in *D. immitis*-infected dogs and their changes following adulticide treatment. Therefore, the objective of this study was to determine if specific radiographic findings are linked to the severity of PH in naturally infected dogs before and after adulticide treatment.

## 2. Materials and Methods

### 2.1. Enrollment and Treatment of the Dogs

This research included 52 dogs owned by clients, which were taken to the Veterinary Medicine Service of the Veterinary Teaching Hospital of the University of Las Palmas de Gran Canaria (Spain). These dogs resided in an area with a high prevalence of heartworm [19,20,21] and were selected for this study based on a positive test result for circulating *D. immitis* antigens (Urano test Dirofilaria^®^, Urano Vet SL, Barcelona, Spain). Clinical history and information of each animal, such as age, sex, and breed, were recorded. A thorough medical history and examination were conducted for each dog to rule out the presence of other diseases that could impact the results. Any animal that was given medication for cardiovascular conditions was not included in this research. Likewise, dogs showing symptoms of heart disease (such as valvular heart disease, cardiomyopathy, and congenital defects) were not included in this research. Diagnostic imaging tests (thoracic radiography and echocardiography), as well as physical examination, clinical history, and anamnesis, were also used to rule out other coexisting cardiorespiratory conditions.

The infected canines underwent adulticide treatment as per the treatment protocol advised by the international Heartworm Societies, incorporating the recently published modifications [22,23,24]. To summarize, upon diagnosis on day 0, the dog started doxycycline administration (10 mg/kg BID) for 4 weeks for the treatment of the endosymbiont bacteria *Wolbachia pipientis*, along with monthly oral tablets for heartworm prevention containing ivermectin (≥6 mcg/kg) and pyrantel pamoate (≥5 mg/kg). On days 30, 60, and 61, the dog received intramuscular melarsomine doses (2.5 mg/kg). A follow-up examination on day 90 determined discharge eligibility based on the absence of abnormalities (adult parasites in echocardiography, radiographic irregularities, or cardiorespiratory symptoms). After 6 months from discharge, on day 270, adulticidal efficacy was confirmed through an antigen detection test. It was advised to restrict exercise during the treatment period, especially from the first melarsomine dose until discharge.

### 2.2. Radiograph and Ultrasound Evaluations

On day 0 (diagnosis), day 90 (discharge), and 270 (6 months after discharge), thoracic radiographs were digitally captured using an RX generator (HFQ 300 P, Bennett, NC, USA) during peak inspiration, without sedation. The radiographic parameters (kVP and mAs) were adjusted individually for each dog based on thoracic thickness. Both right laterolateral and dorsoventral views were taken. Vertebral Heart Size (VHS) was determined following the guidelines previously established by Buchanan and Bücheler [25]. While potential dorsal spine alterations were not a basis for exclusion in VHS interpretation, none of the dogs in this study exhibited such alterations [26]. Furthermore, the CrPA/R4 ratio was calculated based on the measurement of the diameter of the right cranial pulmonary artery (CrPA) passing through the fourth rib (R4) in the laterolateral view, as well as the diameter of R4 just distal to the spine [16,27]. Finally, the CdPA/R9 ratio was determined in the dorsoventral view by the measurement of the diameter of the right caudal pulmonary artery (CdPA) overlapping the ninth rib (R9), in accordance with established guidelines and previous studies [16,27]. All measurements were conducted using electronic callipers on a DICOM workstation (DAIPACS, version 2.71) by a researcher who was unaware of the clinical status of the dogs involved in this study.

The canines also underwent echocardiographic evaluations on days 0, 90, and 270 utilizing an ultrasound device equipped with spectral and color Doppler as well as multifrequency probes (5.5–10 MHz) (Logic P5, General Electric, New York, NY, USA) to determine the presence or absence of PH, in accordance with the guidelines set by the American College of Veterinary Internal Medicine (ACVIM) [11]. The dogs were positioned in right laterolateral recumbence with the transducer placed in the third intercostal space. They remained conscious and were continuously monitored electrocardiographically. Each measurement involved recording six consecutive cardiac cycles, and all echocardiographic assessments were performed by the same researcher. To the aims of this study, the Right Pulmonary Artery Distensibility Index (RPAD Index) was utilized for comparison with thoracic radiographic measurements, with dogs being more likely to exhibit moderate to severe PH if their RPAD Index was <29.5%, a criterion that has been described and validated in dogs affected by heartworm disease [28,29,30]. The dogs were divided into 2 groups: Group A included dogs without PH, and Group B included dogs with PH. All dogs with PH showed a RPAD Index < 29.5%, and all dogs without PH showed a RPAD Index > 29.5%.

### 2.3. Statistical Analysis

The data were analyzed using the SPSS Base 29.0 software for Windows (SPSS Inc./IBM, Chicago, IL, USA). A Shapiro–Wilk test was performed to verify the normal distribution of the data. Additionally, the Siegel–Tukey test was performed to verify the variability of variances between groups. Continuous variables were expressed as mean ± standard deviation. Qualitative variables are expressed as percentages. The non-parametric test of Wilcoxon was used to determine the differences in the different stages (day 0, 90, and 270). In addition, a U–Mann–Whitney test was used to determine differences between dogs with and without PH. In all cases, a *p*-value < 0.05 was determined as significant. Furthermore, Cohen’s D was employed to interpret the differential magnitude between the studied statistical groups. Considering a statistical difference with values > 0.70 for this study.

All dog owners gave their approval for their pets to take part in this research, and an informed consent was specifically signed for this purpose. This study was carried out in compliance with the existing animal welfare laws in Europe.

## 3. Results

Of the dogs studied, 57.7% (30/52) were males and 42.3% (22/52) were females, ranging in age from 1 to 14.5 years (mean: 5.5 ± 0.4 years). Regarding breed, 75% (39/52) were mixed-breed dogs, and 25% (13/52) were purebred. Of the latter, PH was present in the following breeds: Labrador Retriever (*n* = 5), American Pit Bull Terrier (*n* = 1), and Rat Terrier (*n* = 1). On the other hand, PH was absent in the following purebred dogs: Garafian Shepherd (*n* = 2), Canarian Mastiff (*n* = 1), Spanish Water Dog (*n* = 1), Miniature Pinscher (*n* = 1), and Rat Terrier (*n* = 1). Regarding sex, 42.3% (22/52) were females and 57.7% (30/52) were males. In addition, the mean weight of the animals included in this study was 15.33 ± 1.35 kg. On day 0, 40.4% (21/52) of the dogs had PH with a mean RPAD Index of 17%, whereas 59.6% (31/52) were normotensive with a mean RPAD Index of 42%.

Symptoms were observed in 19.35% of dogs without PH, whereas all dogs diagnosed with PH exhibited symptoms, with cough and dyspnea being the most common (Table 1).

The Wilcoxon-signed rank test was used to compare the differences in scores between patients on days 0, 90, and 270. For the RPAD Index, only significant differences were observed between days 0 and 90 (*p* = 0.011). For VHS, statistically significant differences were observed between day 0 and day 90 (*p* = 0.001) and between day 0 and day 270 (*p* = 0.000). However, no statistically significant differences were observed between the measurements at the three time points for the CrPA/R4 and CdPA/R9 ratios (Table 2).

As described in the Section 2, the dogs were further divided into two groups: Group A included dogs without PH (*n* = 31), and Group B included dogs with PH (*n* = 21).

The results showed that RPAD Index values were significantly lower in the group of dogs with PH at all time points (*p* = 2.1218 × 10^−14^ for day 0, *p* = 2.1218 × 10^−14^ for day 90, and *p* = 1.4216 × 10^−12^ for day 270). In addition, while the RPAD Index increased significantly between days 0 and 90 in dogs from group A, the RPAD Index did not change significantly throughout the treatment in dogs with PH (Table 3). Additionally, a dog in group A experienced a decline in pulmonary arterial values, leading to the end of treatment with the presence of PH and an RPAD Index of 22%.

Regarding the radiographic indices, the mean VHS for each group on days 0, 90, and 270 are shown in Table 3. These results indicated that the mean VHS was significantly higher in Group B than in Group A at all time points (*p* = 0.03645206 for day 0, *p* = 0.0131386 for day 90, and *p* = 0.01618378 for day 270). In addition, a significant increase in VHS was observed between days 0 and 270 for Group A dogs and between days 0–90 and days 0–270 for Group B dogs. These results show that VHS tended to increase over time from day 0 to 6 months after treatment, particularly in the group of hypertensive dogs. When compared with the established reference values [25], the number of dogs in Group B exceeding the reference range increased towards the end of this study, whereas the number of dogs in Group A exceeding the reference value remained stable throughout this study (Table 4).

The CrPA/R4 and CdPA/R9 ratios were significantly higher in Group B dogs at all time points (CrPA/R4: *p* = 2.5007 × 10^−8^ for day 0, *p* = 2.5007 × 10^−8^ for day 90, and *p* = 7.5505 × 10^−8^ for day 270; CdPA/R9: *p* = 2.0504 × 10^−9^ for day 0, *p* = 5.7586 × 10^−11^ for day 90, and *p* = 1.4513 × 10^−11^ for day 270). In addition, the CrPA/R4 and CdPA/R9 ratios remained constant without significant variation throughout the treatment in dogs from Group A; however, significant modifications were observed in dogs from Group B during adulticide treatment, which were statistically significant between days 0 and 90 and tended to increase during this study (Table 3).

Cohen’s D, a standardized measure of effect size, was used to interpret the magnitude of differences between the statistical groups studied. For VHS, the calculated Cohen’s D value was between 20.99 and 21.29, indicating a substantial difference between normotensive and hypertensive dogs. This extremely large effect size suggests a clinically meaningful distinction between the two groups in terms of VHS measurements. Similarly, for the CrPA/R4 ratio, the Cohen’s D value was between 2.87 and 2.89, indicating a significant difference between the normotensive and hypertensive groups. Although this was slightly smaller than the effect size for VHS and the CdPA/R9 ratio, it was still a large effect size, indicating a notable difference in the CRPA/R4 ratio between the two groups. In the case of the CdPA/R9 ratio, the Cohen’s D value was between 3.08 and 3.15, while indicating a smaller effect size compared to VHS, still reflected a moderate difference between the normotensive and hypertensive groups, remaining a relevant and potentially informative parameter to discriminate between these groups, underscoring its importance in the assessment of PH (Table 5).

## 4. Discussion

Several studies have suggested that adult *D. immitis* parasites begin to produce lesions once they reach the pulmonary arteries, resulting in proliferative endarteritis that chronically leads to PH and heart failure [3]. PH is therefore a lesion associated with proliferative endarteritis and is consequently common in this disease.

The determination of PH can be indirect, as its direct estimation by right heart catheterization is neither clinically nor practically feasible [31]. Therefore, echocardiography is considered the best method of measurement. Furthermore, previous studies have shown that thoracic radiographs are useful in determining the presence and severity of lesions associated with PH, both in heartworm infection and in other cardiopulmonary pathologies [2,32,33], and some studies have even characterized the association between some radiological changes and echocardiographic findings in dogs with PH [12,13]. As in the present study, these authors reported the presence of cardiomegaly and enlarged pulmonary arteries, among other findings. Recently, a study showed that the assessment of VHS, CrPA/R4, and CdPA/R9 ratios may be used objectively in the preliminary evaluation of chest radiographs of dogs with PH as a screening tool when deciding whether to perform additional tests to assess the presence of PH [16]. The results of the present study confirmed the findings of the previous study, as statistically significant increases in the parameters evaluated (VHS, CrPA/R4, and CdPA/R9 ratios) were observed at all time points between dogs with and without PH.

Furthermore, other previous studies using echocardiography and specific serum biomarkers (i.e., endothelin-1 and acute phase proteins) have shown that PH persisted in dogs after the end of adulticide treatment, at least 10 months after the last dose of melarsomine, reporting that proliferative endarteritis may not be reversible and may persist even after the elimination of adult parasites, thus being a chronic problem that may affect the quality of life and life expectancy of the dog [8,17,18]. Therefore, its study and early detection should be a priority. The results of this study would add to this evidence by demonstrating the persistence of PH, in this case by means of a thoracic radiographic study.

Regarding radiographic indices, the results show an increase in VHS in dogs with PH throughout this study. These results are similar to those reported by other authors who have suggested that heartworm-infected dogs often have an enlarged cardiac silhouette on thoracic radiographs, which is a reliable parameter to discriminate severe PH from non-pulmonary hypertensive dogs [34]. When compared to the reference values [25], 28.6% of dogs with PH had a VHS above the reference value on day 0, rising to 47.6% by the end of this study. Chronic persistence of PH eventually leads to right-sided heart disease, and the observed increase in cardiac silhouette in dogs with PH, despite parasite clearance, may be due to this persistence [5,9]. On the other hand, 12.9% of dogs without PH were observed to have increased VHS on day 0, which remained constant throughout this study. These dogs did not have any other associated pathology, as this was an exclusion criterion, and the animals were examined prior to inclusion in this study. Therefore, this could be due to the use of a generic reference value established without taking into account breed-specific indices, and some dog breeds have variations in VHS reference values [35,36].

Similarly, a significant increase in CrPA/R4 and CdPA/R9 ratios was observed throughout this study, indicating a slight worsening of arterial damage. This is consistent with previous reports by other authors, indicating persistence of endarteritis in dogs after parasite clearance [8,17]. In this research, even one of the dogs went from having normal pulmonary arterial pressure to being hypertensive at the end of the treatment, which had also been observed in a previous study [8]. Furthermore, as mentioned before, a study observed a possible persistence of inflammation at the vascular level by serological detection of acute phase proteins and endothelin-1 in dogs with PH, 7 months after the end of adulticidal treatment [18]. In this context, the results of the present study are in agreement with previous reports, showing that CrPA/R4 and CdPA/R9 ratios could be used to determine the persistence of PH after the end of adulticide treatment.

Given the accessibility of radiological techniques to veterinary clinicians, the low technical requirements, and the low cost, the results of this study are of great interest as an aid in the post-treatment evaluation of dogs with heartworm. Given the high incidence of PH in dogs with *D. immitis* and the persistence of endarteritis and PH in chronically infected dogs, the objective determination of VHS, CrPA/R4, and CdPA/R9 ratios may be useful for long-term monitoring when other imaging tools are not available or in support of other imaging tools. This could have a beneficial impact on the animal, as it would allow for close monitoring, leading to better quality and life expectancy.

## 5. Conclusions

In conclusion, knowledge of the response and possible changes in the pulmonary vasculature after adulticide treatment by radiological assessment of VHS, CrPA/R4, and CdPA/R9 ratios could be useful in the detection and monitoring of PH in dogs during adulticide treatment and for close monitoring thereafter.

## Figures and Tables

**Table 1:** Distribution of symptoms and frequency by groups. Group A: dogs without pulmonary hypertension. Group B: dogs with pulmonary hypertension.

Groups	Symptoms	Percentage
Group A	Asymptomatic	80.65% (25/31)
	Cough	19.35% (6/31)
Group B	Asymptomatic	0% (0/21)
	Cough	95.24% (20/21)
	Dyspnea	57.14 (12/21)
	Weight loss	4.76% (1/21)

**Table 2:** Results of radiographic measures in all studied dogs in different time points. RPAD Index (Right Pulmonary Artery Distensibility Index); VHS (Vertebral Heart Size); CrPA/R4 (right cranial pulmonary artery passing through the fourth rib in the laterolateral projection ratio); CdPA/R9 (right caudal pulmonary artery to the ninth rib in the dorsoventral projection ratio). (*): *p* < 0.05.

Measure	Time Point	*p*-Value
RPAD Index	Day 0–Day 90	0.01108686 *
	Day 0–Day 270	0.37772975
	Day 90–Day 270	0.14238119
VHS	Day 0–Day 90	0.00137051 *
	Day 0–Day 270	0.00027207 *
	Day 90–Day 270	0.05733536
CrPA/R4	Day 0–Day 90	0.16833147
	Day 0–Day 270	0.2023426
	Day 90–Day 270	0.36260403
CdPA/R9	Day 0–Day 90	0.16833147
	Day 0–Day 270	0.22349953
	Day 90–Day 270	0.18534784

**Table 3:** Results of the Wilcoxon test for all measures by time points and groups of studied dogs. RPAD Index (Right Pulmonary Artery Distensibility Index); VHS (Vertebral Heart Size); CrPA/R4 (right cranial pulmonary artery passing through the fourth rib in the laterolateral projection ratio); CdPA/R9 (right caudal pulmonary artery to the ninth rib in the dorsoventral projection ratio). Group A: dogs without pulmonary hypertension. Group B: dogs with pulmonary hypertension. Results are displayed as mean ± standard deviation. NS: No significant differences were found between time points. (*): *p* < 0.05.

Measure	Group	Day 0	Day 90	Day 270	Time Points with Significant Differences between Measures	*p* Value
RPAD Index	Group A	42% ± 0.06	43.16% ± 0.07	42% ± 0.07	Day 0–Day 90	0.043641 *
	Group B	17% ± 0.11	17.96% ± 0.11	17.84% ± 0.11	NS	>0.05
VHS	Group A	9.85 ± 0.74	9.92 ± 0.68	9.98 ± 0.72	Day 0–Day 270	0.01313415 *
	Group B	10.29 ± 0.74	10.43 ± 0.76	10.48 ± 0.76	Day 0–Day 90	0.01844421 *
					Day 0–Day 270	0.01313415 *
CrPA/R4	Group A	1.08 ± 0.16	1.06 ± 0.18	1.06 ± 0.16	NS	>0.05
	Group B	1.54 ± 0.36	1.59 ± 0.41	1.60 ± 0.47	Day 0–Day 90	0.01448255 *
					Day 0–Day 270	0.04484749 *
CdPA/R9	Group A	1.14 ± 0.15	1.13 ± 0.16	1.12 ± 0.15	NS	>0.05
	Group B	1.68 ± 0.38	1.77 ± 0.39	1.79 ± 0.39	Day 0–Day 90	0.02499993 *
					Day 0–Day 270	0.01999474 *

**Table 4:** Results for Vertebral Heart Score (VHS) results are distributed by time points in the studied dogs. Group A: dogs without pulmonary hypertension. Group B: dogs with pulmonary hypertension. Results are displayed as mean ± standard deviation.

	Groups	VHS	Dogs above Reference Value (≥10.5) [25]
Day 0	GROUP A	9.85 ± 0.74	12.90% (4/31)
	GROUP B	10.29 ± 0.74	28.57% (6/21)
Day 90	GROUP A	9.92 ± 0.68	12.90% (4/31)
	GROUP B	10.43 ± 0.76	42.86% (9/21)
Day 270	GROUP A	9.98 ± 0.72	12.90% (4/31)
	GROUP B	10.48 ± 0.76	47.62% (10/21)

**Table 5:** Cohen’s D for the studied parameters. RPAD Index (Right Pulmonary Artery Distensibility Index); VHS (Vertebral Heart Size); CrPA/R4 (right cranial pulmonary artery passing through the fourth rib in the laterolateral projection ratio); CdPA/R9 (right caudal pulmonary artery to the ninth rib in the dorsoventral projection ratio).

Measure	Cohen’s D	Value	Interpretation
VHS	Day 0	20.9929921	Large effect
	Day 90	21.1714818	Large effect
	Day 270	21.2935957	Large effect
CrPA/R4	Day 0	2.86616835	Large effect
	Day 90	2.88762566	Large effect
	Day 270	2.84047934	Large effect
CdPA/R9	Day 0	3.07601672	Large effect
	Day 90	3.15304145	Large effect
	Day 270	3.11923901	Large effect

Cohen’s D: d = 0.2 small effect, d = 0.5 moderate effect; d = 0.8 large effect.

## Data Availability

All data generated or analyzed during this study are included in this article. The datasets used and/or analyzed during the present study are available from the corresponding author upon reasonable request.
